# Topics of Public Interest

**Published:** 1912-11

**Authors:** 


### TOPICS OF PUBLIC INTEREST.

The cornerstone of Mt. St.Mary Hospital, Niagara Falls, was laid on Monday, Sept. 30, Bishop Colton officiating.

22 Medical Schools have been disqualified by the Regents of N. Y. State, as not conforming to the present requirements. Graduates of these institutions, however, may establish their right to a license by passing a special examination.

---

Examination for Appointment to the Public Health Service. Candidates for admission to the grade of assistant surgeon will be examined at the Bureau of the Service, 3 B St., S. E., Washington, D. C., Monday, Nov. 11, at 10 A. M. Candidates must be between 22 and 32, must furnish testimonials as to character, must have had one year's interne service or two years' practice. The salary is $2,000 with quarters or allowance of $30 per month. The salary is increased by promotion and term of service, so as to aggregate about $4,500 at the end of fifteen years.

---

Recent Appointments, Buffalo Health Department. Dr. D. V. McClure has been transferred from the medical school examinership to be Physical Examiner in the Bureau of Child Hygiene at a salary of $2,000.

Dr. Joseph C. O'Gorman has been promoted from Milk Examiner to Dr. McClure's former position, at a salary of $1,200.

The position of Secretary to the Assistant Health Commissioner has been established, with a salary of $900. This position will be filled by Miss Flavilla Wende, daughter of the late Health Commissioner, Ernest Wende, M. D.

Miss Josephine C. Lynch has been appointed female attendant in the Bureau of Child Hygiene at a salary of $720.

Dr. James H. Donnelly has been appointed Tuberculosis Inspector, vice Dr. Otto R. Eichel, resigned, at a salary of $1,000.

Dr. Edward P. Forrestel has been appointed assistant medical school examiner.

But, why not pay the City Physicians adequate salaries also?

---

Better Light for Night Schools is recommended by Drs. F. Parks Lewis, H. W. Cowper and Frank Farrell, committee of investigation for the Buffalo public schools. This reminds us that, in the old days, the boys entering the Central High School were seated in the dark corner. This was a natural courtesy to the senior classes and proper enough, although the tissues of the incoming class were most sensitive. And it must be admitted that the lenses which were required during the next year or so gave us a more intellectual appearance and are very convenient in keeping dust out of the eyes.

Lady Albina Brodrick is visiting America to study nursing methods mainly as expounded by Adelaide Nutting of Columbia. Lady Brodrick will apply the information gained, as well as the money which she hopes to raise, in the interests of the Irish Hospital in County Kerry. For some reason, our Irish citizens are most loyal to the mother country, especially after two or three generations, than any others. We have in mind a family of enthusiastic Irish girls and boys. They and their parents were born in America; one parent is German, the other is Scotch-Irish. In contrast to such sentiment, the following incident occurs to us. Last spring, at Albany, one of the prominent old doctors was speaking of the fewness of years remaining to him. We asked him if he did not ever wish to go home. "Oh, yes," he replied, "I am going to take the 9:45 train tomorrow morning." When we made it clear that we referred to his birthplace, he made it equally clear that he had no love for his motherland, and that he was purely an American.

---

INCREASED REQUIREMENTS IN MEDICAL EDUCATION. Next year, matriculants in N. Y. State will be required to have had one year of college training in specified studies.

---

HIGHER DEATH RATE NEEDED. In the U. S. three out of four murderers are not brought to trial and only one in a hundred man-killers is killed. In Germany, the chances of punishment are said to be 95 per cent. Without discussing the pros and cons of capital punishment, it is obvious that the relative safety of murderers favors murder. It has been stated that the mortality rate of murderers is far less than that of railroad switchmen. In 1911, there were 119 deliberate murders in New York, while in London, where poverty of desperate degree is far more prevalent, there were only 19.

The *Buffalo Progressive* of Oct. 10, 1912, lists 10 accidental deaths, 3 murders and 4 suicides for the city, for the week preceding. The total average number of deaths per week for Buffalo should be about 140. Ordinarily—we were about to say normally—one death in 10 is by violence. No one charges the medical profession with the responsibility for violent deaths directly, but, whenever gross death rates and failures to secure ideal average longevity are considered, the onus is placed on our profession and few make even the 10 per cent. allowance.

---

SCHOOL INSPECTION. Out of 49,764 children examined in Buffalo in the last two years, 19,456 had defective teeth, 6,563 hypertrophied tonsils, 3,959 defects of vision. Health Commissioner Fronczak is using these statistics as an argument for the appointmnt of dental inspectors and establishment of dispensaries.

---

Increased Salaries Wanted. Among other requests for salary increases, under consideration by the Buffalo common council, are the following: Medical Examiner, $3,500 to $4,200; Assistant, $2,500 to $3,000. As Dr. Jekyl, we are most emphatically in favor of dental inspectors and dispensaries and of raising professional salaries, but we must confess that as Mr. Hyde, paying from 15 to 30 per cent of the gross receipts of property in city taxes, we can appreciate the narrow-minded stinginess of many.

---

Increasing Accident Reports. The N. Y. Association for Labor Legislation (Paul Kennaday, Sec'y., 131 E. 23d St., N. Y.,) reports 19,567 industrial accidents for the quarter ending Sept. 30; 13,731 occurred in factories and 5,628 in building and engineering work. The former are about 1,000, the latter about 3,000 more than for the corresponding quarter year of 1911, indicating probably not so much actual increase as more thorough reporting and, probably, a change of classification. The Department of Labor, even with its increased staff, is unable to maintain an adequate system of inspection. The fact that the accident rate, as reported, doubtless with many omissions, especially of minor accidents, is nearly 1 per cent for the whole population, regardless of age or sex or occupation, shows that the problem needs more thorough attention. Remember these items, as well as the automobiles, railroads, homicides and others, when some orator, basing his remarks on general disease and mortality rates, is telling how little medical science has accomplished, after all.

---

College of Medicine of Syracuse University. The trustees announce that Professor Leverett Dale Bristoll, A. B., Wesleyan, M. D., Johns Hopkins, has been called from Minneapolis as professor of bacteriology. Also the election of Earl V. Sweet, A. B., Colgate, M. D., Cornell, as Instructor in Histology.

The election of Mr. John R. Rice, B. S. Wesleyan, to be instructor in the Department of Hygiene and Preventive Medicine and Assistant in the Municipal Laboratory.

The election of Albert G. Swift, M. D. Syracuse University of New York City to be instructor in Clinical Surgery.

The election of John W. Cox, M. D., Syracuse University, to be instructor in Pathology.

THE NEW YORK POST-GRADUATE SCHOOL AND HOSPITAL has recently been re-organized. Dr. James F. McKernon has been elected president in place of Dr. George N. Miller, resigned. The officers now are: President, James F. McKernon, M. D.; Second Vice-President, Edward Quintard, M. D.; Treasurer, William Fahnestock, Esq.; Secretary, Arthur F. Chace, M. D.; Secretary of the Faculty, George G. Ward, Jr., M. D.; Superintendent, H. T. Summersgill, M. D.

---

A DETERMINED effort is being made to secure the combination of three Baltimore medical colleges, viz.: the University of Maryland, the College of Physicians and Surgeons, and the Baltimore Medical College. In order to insure the success of such a plan, it is thought that an endowment fund of $500,000 to $1,000,000 should be raised.

---

THE MEDICAL COLLEGES of the State of New York, acting under the direction of Commissioner Porter of the State Health Department, are arranging a systematic course of instruction in public health and the prevention of disease. The plan of instruction is being prepared by the following committee: Dr. William A. Howe, deputy state commissioner of health; Dr. John L. Heffron, Syracuse University College of Medicine; Dr. Royal S. Copeland, of the New York Homeopathic Medical College; Dr. Bensel, of the Medical Department of Columbia University; and Dr. W. H. Park, of the Bellevue and New York University Medical College.

---

THE BELL TELEPHONE SERVICE IN BUFFALO HAS DISCONTINUED ITS MORNING CALL so that physicians having to take early trains will have to depend upon the Frontier or upon an alarm clock. We recommend a rachet-and-cog system for public service legislation so as to secure a movement only in the direction of improved service and lessened cost of any public utility.

---

MUNICIPAL TUBERCULOSIS HOSPITAL. The medical committee has decided to advise the erection of an administration building in the immediate future, the hospital proper to be built in two or three years. Meanwhile, the supervisors are considering a temporary addition to the Erie County Hospital, which is overcrowded.

---

CONDEMNATION OF MEDICAL NEWS. The Baltimore (Maryland) County Medical Association has passed a resolution (1) condemning the publication of patients' names, diseases or conditions requiring operation, and names of attendants and of hospital, in the daily press, as contrary to the principle of professional secrecy; (2) establishing a list of offenders and pledging the members not to consult with them nor to use the hospitals. While agreeing with the general spirit of these resolutions, it must be admitted that in most cases the publication of patients' names and diseases or conditions requiring operation, as well as the fact that the patient is treated at a given hospital, is not contrary to the wishes of patients and their families, that such matters are strictly news when prominent persons are concerned and that for persons in private life, the notification is a convenience to friends. It is rather an extension of the code of ethics to demand that the name of a hospital shall not be publicly mentioned and we, personally, do not intend to ostracise any member of the profession because his name is mentioned in connection with a case, unless it appears that he is responsible for the item and is deliberately seeking advertisement.

---

Anterior Poliomyelitis. The Buffalo epidemic began with 2 cases in January; there being no others till 1 occurred in May. In June there were 3, in July 62 with 4 deaths, in August 124 with 10 deaths; in September 86 with 13 deaths, a total of 278 cases and 33 deaths for nine months. In October, there were 30 cases and 8 deaths. Dr. W. J. Rosenau, of the U. S. M. H. and P. H. Service, a classmate of the editor's at the University of Pennsylvania, and appointed with him to the service, has recently reported the transmission of the disease from monkey to monkey by the common fly, stomoxys calcitrans, six of twelve healthy monkeys contracting the disease.

---

Death of Millie-Christine. This two-headed and four-legged woman, or rather these twins, for the two heads had distinct personalities and did not even sleep at the same time necessarily, have recently died, the plural being shown to be more appropriate by the death of one head several days before the other. The twins were negresses, born in slavery and once sold for $40,-000. Aside from the teratologic interest in the case, one can not avoid expressing sympathy for the terrible ordeal of the second twin, after the death of the sister head and while awaiting a certain doom under the most shocking conditions.

---

The Nobel Prize for the most noteworthy achievement in medicine during the current year was awarded on Oct. 10, 1912, at Stockholm, to Dr. Alexis Carrel of the Rockefeller Institute for his work in the suture of blood vessels and transplantation of organs. The prize was $39,000. Dr. Carrel was born in

France in 1873, received the medical doctorate in 1900 and came to America in 1903.

---

THE NIAGARA FALLS WATERWORKS have been completed at a cost of over a million. About 44 miles of new mains and a filtration plant are the most expensive items. Typhoid is said already to have shown a decline.

---

NEW FACTORY LAWS in New York State, limit the working hours of women to 9 a day and 54 a week, and provide that no woman shall be employed within four weeks after giving birth to a child. Children will not be allowed to work in factories unless over 14 years and with a certificate of health. The Commissioner of Labor is also authorized to close factories not clean and sanitary and not maintaining proper toilet facilities, including hot and cold water and individual towels, whenever the industry involves poisonous dust, gases and fumes. Further legislation is contemplated. We venture to suggest that labor laws and educational regulations should include a provision for absence from duty for one or two days a month, on certificate of necessity by a physician, in the case of any woman or girl. So far as schools are concerned, it should be provided that such absence should not detract in any way from class standing.

---

NURSE FOR POST-PARALYTIC CASES. The Buffalo Department of Health has engaged a nurse to give massage and other care to cases of infantile paralysis. She will work exclusively under the direction of the attending physician for cases deserving charity.

---

THE MEDICAL DEPARTMENT OF THE UNIVERSITY OF BUFFALO has registered 59 students in the Freshmman class, beside several special students. A wholesome sign is the fact that the four classes are nearly equal in numbers, allowing for the natural weeding out process and the unprecedentedly large registration last year. It is gratifying, also, to note that alumni are sending their brothers and sons to their alma mater.

---

EXAMINATION FOR MINE SURGEONS.—The U. S. Civil Service Commission announces an examination on Nov. 20, at various places. One vacancy exists at present. The salary ranges from $2,000 to $3,000. Application should be made to the Commissioner at Washington, or to the Secretary of the Board of Examination at the following places in New York State. (C. H. means Custom House.) Binghamton, Buffalo, Elmira, Ithaca, Jamestown, New York, C. H., Ogdensburg, C. H., Plattsburg, C. H., Poughkeepsie, Rochester, Syracuse, Troy and Utica.

